# Understanding Korean and the U.S. Custodial Grandparents Through the Lens of Role Theory

**DOI:** 10.1093/geroni/igaf122.888

**Published:** 2025-12-31

**Authors:** Youjung Lee, Nancy Mendoza

**Affiliations:** Binghamton University, Binghamton, New York, United States; The Ohio State University, Columbus, Ohio, United States

## Abstract

This study explored grandparent caregiving experiences during and post-pandemic in Korea and the United States. Using a phenomenological approach, semi-structured interviews were conducted with 20 custodial grandparents—10 from each country. Among the Korean grandparents, the mean age was 73 years, two were grandfathers, and on average, they had been raising 1.4 grandchildren for 6 years. Reasons for raising their grandchildren included divorce (7 instances), death (1), substance abuse (1), or financial hardship (1) of the grandchildren’s parent(s). U.S. interviewees identified as female (10), White (9) or African American (1) and had a mean age of 59 years. On average, they had been raising 1.8 grandchildren for 8.1 years. Reasons for grandparents raising grandchildren included parents’ substance abuse (7), parent’s leaving grandchildren after a divorce/relocation (2), or supporting a single parent (the grandparent’s child (1). Under the guidance of role theory, the caregivers’ experiences were explained by themes of role consensus, role conformity, and role conflict. Despite Korean and U.S. grandparent groups performing a similar role, relative differences were observed, including Korean grandparents with increased role conformity and U.S. grandparents with increased role conflict. The distinction highlights the importance of understanding grandparent caregiving in cultural contexts and ensuring that interventions are culturally responsive. Regardless of cultural context, the grandparent caregivers disclosed relatively positive experiences of the pandemic, testifying to their resilience. Lastly, critical role supporters were identified at various levels, demonstrating that developing support systems can be a key in empowering grandparent caregivers.

